# Social Capital Resources in Coping with Distance Education during the COVID-19 Pandemic: A Content Analysis of the Statements of Teachers Working in Poland at Different Educational Stages

**DOI:** 10.3390/ijerph19073905

**Published:** 2022-03-25

**Authors:** Sylwia Jaskulska, Barbara Jankowiak, Emilia Soroko

**Affiliations:** 1 Faculty of Educational Studies, Adam Mickiewicz University, 61-712 Poznan, Poland; barbara.jankowiak@amu.edu.pl; 2 Faculty of Psychology and Cognitive Science, Adam Mickiewicz University, 61-712 Poznan, Poland; emilia.soroko@amu.edu.pl

**Keywords:** social capital, school social capital, teachers, distance education, COVID-19 pandemic

## Abstract

The article aims to show social capital resources in coping with distance education during the COVID-19 pandemic of Polish teachers working at different educational stages. The sample consisted of 1104 women (91.2%) and 107 men (8.8%) who described their remote professional experiences as valued positively during the pandemic. The collected verbal material was analyzed with quantitative content analysis based on theory-driven categories of social capital: Relationships, trust, commitment, and fulfilling obligations. Then, the frequency of words belonging to the categories in each participant’s utterance was assessed. The results indicate that when describing positive experiences (situations and events) during distance education, teachers referred mainly to social capital resources in terms of relationships and fulfilling obligations. The results indicate that teachers working in secondary schools in Poland put less emphasis on building social capital during distance education, especially in terms of relationships, than teachers working with younger children.

## 1. Introduction

Social capital theories have their origins in economics. The constituting idea is related to the belief that apart from material capital, people also have other forms of capital. Despite their non-material nature, social, cultural, or human capital can be considered as material or economic. This means that one can talk about the size of these assets and measure them. An important feature of all types of capital is that conversions from one type to another are possible [1]. This means that the high assets of one type of capital are related to the high resources of others. Therefore, when researching the social capital of a community, one can infer, for example, how this community will cope with a difficult situation, or to identify the possible directions of its development—and with this approach to also determine possibilities to multiply the resources of various types of capital [2,3].

We define social capital, following Putnam [4] and Fukuyama [5], as a resource owned by an individual entire community. In Putnam’s [4] definition, social capital comprises moral obligations and norms, social values (especially trust), and social networks (especially voluntary associations) that facilitate co-operation and mutually supportive relations in communities and nations. Fukuyama [5] points out that social capital is the existence of a certain set of informal values or norms shared among members of a group that permits cooperation. Therefore, a community with high social capital assets is characterized by a high quality of relations and sharing of bonding norms. Authors dealing with social capital pay special attention to trust and commitment as important manifestations of this type of capital [6].

Social capital is essential in social research because its ample resources are indicators of, for example, ways of dealing with difficult situations as well as the strength and development of communities [7]. The theory of social capital is present also in educational research [2,8]. Research results indicate that school social capital predicts lower school burnout and better academic achievement among students [9] and teachers’ job satisfaction [10].

Research on the educational dimension of social capital is being conducted in Poland. Results indicate, for example, the existence of the phenomenon of capital translation in the educational field. The higher the social capital resources of primary and secondary schools, the better the students’ university experiences [11,12]. Such a tendency found in such studies is consistent with treating social capital resources as an indicator of social inequality. The more capital a student has at the beginning of education, the better the schools he/she attends and the more resources he/she has because these schools create conditions for their multiplication [13,14]. Thus, some studies show individual educational paths in terms of social capital, but no studies to date show how schools at different levels differ in this respect. There is a particular lack of studies covering the earliest stages of education, namely pre-school and early childhood education. However, it is possible to make inferences about the size of the capital at different levels of education by looking at their specifics in creating conditions for building resources such as involvement, trust, and relationships. The research image of the early stages of education allows us to conclude that they are more conducive to relationships or trust. In subsequent stages, the overload in school curricula and pressure on test and examination results dominate [15]. Research conducted in Poland indicates that the school climate is better at lower educational stages. For example, in the TALIS 2013 study, relationships at school are assessed as better by teachers from primary schools than from secondary schools [16]. In addition, research shows that, for example, collegial bonds are increasingly weaker in Poland at successive educational levels and generally weaker than in other countries. For example, according to the results of PISA studies, Polish 15 year olds declare being liked by other students less frequently than their peers in OECD countries [17].

In terms of the resources of various types of capital, they can also apply to teaching experiences. It turns out, for example, that the social capital of teachers starting their professional life is built in relationships with other teachers rather than with the school’s management [18]. Trust is a critical element of school culture and school leadership [19] and the learning outcomes of students [20]. Thus, research on social capital makes it possible to infer the functioning of individuals and groups in everyday education situations.

This study uses social capital theory to analyze Polish teachers’ experiences with distance education during the COVID-19 pandemic. Distance education—understood as situations when learners and what is being learned do not share the same space/time context [21]—was introduced in Poland in March 2020, and it was maintained until June 2021 (with short breaks when students were studying in class, for example, at the beginning of the 2020/2021 school year). Before the COVID-19 pandemic, distance education was uncommon or non-existent in Poland [22]. Changing the teaching methodology from direct contact at school to Internet-mediated communication was challenging for Polish teachers [23]. We use the term distance education with the awareness that it was not a planned form of learning. The term emergency remote teaching (remote solutions are used for teaching that would otherwise be delivered as face-to-face courses and which will revert to that form when the crisis is over [24,25,26] seems to be more suitable, but distance education or learning is commonly used in the context of the situation in Poland during the pandemic, so we also use this term [27,28,29,30].

In Poland, there are the following educational stages in the education system (the higher education stage is not considered in the present study, so it is not addressed) [31]:

- institutions for children aged 0–3 years: Crèche or kids club (not obligatory);
- institutions for children aged 3–6 years: Nursery school or pre-school class in a primary school, pre-school unit, pre-school center (optional for three- to five-year-old children and obligatory for six year olds);
- primary education: Grades 1–3 (early school education) and grades 4–8 (teaching by subject);
- secondary education: Four-year general secondary school, or five-year technical secondary school, or three-year Stage I sectoral vocational school and two-year Stage II sectoral vocational school.

Compulsory education starts in pre-school class in a primary school or nursery school (six-year-old children) and covers primary education.

During the COVID-19 pandemic, pre-school and first-grade children had the shortest durations of distance learning. Older students learned remotely most of the time from March 2020 to June 2021, performing their school obligations. In practice, this involved various forms of synchronous and asynchronous online work. Typically, students and teachers attended the meetings through various enabling platforms. Assignments and student work were also uploaded by using different communication channels [32]. With this change, many new challenges have emerged.

The possibility of implementing the school curriculum, assessing and examining students in these conditions, and building and maintaining educational relationships or new dimensions of school inequalities have become vivid problems in pedagogical research [32,33,34]. First, the school’s focus on the didactic function was diagnosed, along with difficulties in fulfilling the duties of caring and upbringing. The most serious problems include peer relations disturbance, lowered well-being of students, and lack of digital hygiene [35].

The current study aimed to look at teachers’ positive experiences during distance education. We assumed that the encouragement to talk about positive experiences would evoke references to mobilized resources. We were interested in how teachers described their positive work experiences in terms of different aspects of school social capital and the emotional tone of these statements (positive vs. negative). In particular, we investigated whether teachers working at different stages of education differed in the frequency with which they recalled the social capital and emotional valence of the events they narrated. Thus, our aims were both descriptive (exploratory, when we asked how teachers describe their experiences) and explanatory (in a way we expected differences in how they account experiences regarding what stage of education they work at).

When the latter aim is considered, we expected teachers in kindergarten and primary grades 1–3 to emphasize school social capital resources more than teachers in primary grades 4–8 and secondary school. Our hypothesis derives from the fact that, as research indicates, the school social climate is better at lower educational stages, and teachers from primary schools better assess school relationships than teachers from secondary school. Moreover, in Poland, in kindergarten and primary school, in grades 1–3, pupils usually have several years of integrated classes with one teacher, which is conducive to building close, trusting, and committed relationships. In contrast, from grade 4 onwards, subject teaching begins, and most subjects are taught by other teachers, which may hinder close relationships. At the same time, concerning subsequent stages of education with older pupils, the problem of overloaded school curricula, pressure on test results, and examinations, which may hinder the building of social resources, dominates. Suppose that in the free statements (on the positive aspects of remote education during a pandemic) of teachers of kindergarten and grades 1–3 there is more emphasis on different aspects of the school’s social capital than in the case of teachers of older pupils. In that case, it means that they have better access to the resources of this capital. Consequently, they can access these resources and use them in the problematic situation of changing educational work in an epidemiological emergency.

In our approach, the elements of school social capital are relationships, trust, commitment, and fulfilment of obligations. Relationships are understood as mutual interactions among members of the school community that create community bonds [9,36]. Trust relates to positive experiences, friendliness from members of the school community, and support experienced [3,36,37]. Involvement is active participation and motivation to engage members of the school community in joint activities [36]. Fulfilling obligations are forms of behaviour resulting from the social role of members of the school community and thus everyday activities related to teaching and learning [36,38].

Based on a similar assumptions about differences in learning environments between the stages of education, we also expected that the social (relational) potential of the early stages of education would result in higher positive emotional tone (and negative emotional tone lower) in the early stages of education than later.

## 2. Materials and Methods

### 2.1. Participants and the Procedure of Data Collection

The selection for the study group was purposive (male/female remote teachers) and voluntary. The sample consisted of 1104 women (91.2%) and 107 men (8.8%). Teachers were employed in kindergarten (429 persons, 35.4%); primary school, grades 1–3 (113 persons, 9.3%), grades 4–8 (329 persons, 27.2%); and also general secondary school (118 persons, 9.7%), technical secondary school (43 persons, 3.5%), and sectoral vocational school (6 persons, 0.5%). Of the total group, 173 (14.3%) teachers worked in more than one type of school. The seniority of the survey participants was as follows: over 11 years (771 people, 63.7%), 6–10 years (167 people, 13.8%), 2–5 years (184 people, 15.2%), and less than two years (89 people, 7.3%). The questionnaire was sent in May and June 2020 to selected schools and kindergartens in all provinces in Poland and was available on Facebook on the websites of teacher associations.

During both the research planning and implementation we followed the principles stated in the Declaration of Helsinki and also the requirements set out in this journal regarding survey studies.

The questionnaire consisted of a sociodemographic question and an open-ended question and two other scales not referred to in this report. The questionnaire began with the following information: To whom the study was addressed (in-service teachers and remote teachers), the purpose and duration of the study, and the rights of study participants (assurance that the study is anonymous and voluntary, that participants could withdraw from the study at any time, that all responses would be confidential, and that participants could ask questions by e-mail about the role of the researcher). E-mail contact for the investigators was also provided. Participants were then invited to proceed to the questionnaire itself.

The respondents gave a short written statement, preceded by the following instruction, encouraging them to write about positive experiences: ‘There are many difficulties associated with distance education—organisational, technical, methodological—related to contact with parents, male and female students. However, please describe a situation/event that you consider positive. Maybe something like that happened in your relationship with a parent/student? On a didactic area? In the educational sphere?’

The main research questions are: How teachers described their positive work experiences in terms of different aspects of school social capital and the emotional tone of these statements (positive vs. negative)? Whether teachers working at different stages of education differed in the frequency with which they recalled the social capital and emotional valence of the events they narrated? These problems were the framework for analyzing the respondents’ responses.

### 2.2. Content Analysis Procedure

In order to create categories describing social capital, the collected verbal material was subjected to quantitative content analysis based on theory-driven indicators [39,40]. Based on the analysis of the available literature (see theoretical introduction), we concluded that the categories of social capital would be relationships, trust, commitment, and fulfilling obligations.

The unit of analysis was the words used in the utterances by the subjects. The threshold for selection of utterances qualified for content analysis was min. = 3 words, max. = 444 words (M = 23.06, SD = 28.12, skewness = 5.917, kurtosis = 63.52). The first stage of the analysis was to concatenate the whole corpus of utterances into a single set of words (the corpus consisted of words). Then, the frequency of words in this set was analyzed. The list of unique words obtained in this way was used to divide the words into those expressing a connection with a specific category of social capital (e.g., trust). A word’s presence in a given category was determined by the concurrence of two independent competent judges who worked with the list of unique words. The expert judges first independently assigned words to the established social capital categories (relationships, trust, commitment, or fulfilling obligations) and the set ‘other’, comprising words not belonging to these categories. The judges then reached a consensus on the category presence of the words through discussion. A third person then checked each set of words against the categories. This person made sure that each word belonged only to one category (ensuring that the categories were separate) and that words from the ‘other’ set were not accidentally omitted (ensuring that the categories were exhaustive). In this way, we obtained a dictionary for each category, based on the collected material, but theoretically derived from the theory-based classification of types of capital (Table 1).

The second stage was to analyze the frequency of words belonging to the categories in the individual utterances of the subjects. To assess the word occurrences, we used computer-assisted content analysis (CasualConc software 2.0.7, Osaka, Japan) with external dictionaries developed as described above. For each person surveyed, we obtained the frequency of the selected word occurrences (those belonging to the specific dictionary developed for each category), which was then relativized to the number of words used by the person, according to the formula (number of word occurrences from dictionary/number of words × 100). From the specific categories (relations, trust, commitment, fulfilling obligations), we also created an overarching category named the “social capital”. In the same way as above, the third stage of the analysis was to create one additional classification independent of those described, concerning the emotional aspect of the statements (positive and negative words), that allowed us to assess the emotional tone of the teachers’ statements (Table 1).

### 2.3. Measures

In this study, the grouping variable was the educational stages where teachers worked at the moment of the study: (1) Nursery school or pre-school class, (2) primary education (grades 1–3, early school education), primary education (grades 4–8, teaching by subject), (3) secondary education (4-year general secondary school, 5-year technical secondary school, 3-year Stage I sectoral vocational school, 2-year Stage II sectoral vocational school), and (4) having worked in more than one stage simultaneously.

Moreover, there were two different groups of independent variables. (1) The school’s social capital was operationalized by indices derived from theoretical assumptions. Indices were built based on words (tokens) used in written statements about positive experiences in school during the COVID-19 pandemic. Social capital was described on four dimensions: Relationships, trust, commitment, and fulfilling obligations. (2) The emotional tone of the utterances (positive and negative) was examined based on positively and negatively charged words used in the written statements.

## 3. Results

### 3.1. Descriptive Statistics

In the area of social capital resources, we distinguished the following categories: Relationships, trust, commitment, obligation fulfilment, and the area of positive and negative impression (positive and negative categories).

The results indicate that teachers, when describing situations and events that they evaluated positively during remote education, referred in their statements mainly to the resources of social capital in terms of relationships and fulfilment of obligations. We also looked at the emotional character of the statements. Interestingly, even though the question was about positive events against a background of negative ones, there was more emphasis on words referring to negative emotions than to positive ones (Table 2).

Intercorrelations were also examined for exploratory purposes, and statistically significant correlations were noted between the highlighted aspects of school capital and other categories. Statistically significant weak correlations were obtained between commitment and words expressing positive emotions (rho Spearman = 0.2814, *p* < 0.001). In contrast, social capital in the aspect of relationships is negatively associated with the frequency of words expressing negative emotions (rho Spearman = −0.2179, *p* < 0.001) (Table 2).

### 3.2. Social Capital Resources at Different Stages of Education

We examined whether those teachers working at different educational stages differed in the intensity of references to different types of social capital in their statements. We were particularly interested in whether pre-school teachers and teachers of the first stages of education strongly emphasized social capital in their statements—as mentioned above, research show that the school social climate is better at lower educational stages, and teachers from primary schools better assess school relationships than teachers from secondary school. Our expectations were largely confirmed, as specified below.

The results of the analyses indicate that teachers working at different stages of education differ significantly in their social capital resources during the COVID-19 pandemic in terms of the generalized social capital index as well as its specific dimensions such as relationships and commitment (Table 3).

Detailed analysis with post hoc tests showed that there are statistically significant differences in the perception of generalized social capital resources between teachers working in secondary school and teachers of pre-primary education (W = −6.9814; *p* < 0.001), primary school in grades 1–3 (W = −5.2893; *p* < 0.01), primary school in grades 4–8 (W = 5.7628, *p* < 0.001). Greater capital resources are indicated by pre-service teachers (M = 29.71; SD = 17.94), primary school teachers working in grades 1–3 (M = 28.78; SD = 16.74), and those working in grades 4–8 (M = 28.17; SD = 117.13) compared to those working in secondary school (M = 22.10; SD = 15.12) (Table 4).

There are also statistically significant differences in the perception of social capital resources in the area of relationships between teachers working in kindergarten, those working in secondary school (W = −7.4314, *p* < 0.001), and those working at different educational stages simultaneously (W = −5.0114, *p* < 0.01). Social capital in this area appears with greater intensity in the statements of pre-school teachers (M = 14.30; SD = 11.33) than of secondary school teachers (M = 9.898; SD = 10.11) and those working at different educational stages simultaneously (M = 11.95; SD = 12.50) (Table 4).

According to the results, there are statistically significant differences in the building of social capital during distance education in the area of relationships between teachers working in primary school in grades 1–3 and those working in secondary school (W = −5.6878; *p* < 0.001) and working at different educational stages simultaneously (W = −3.9987; *p* < 0.05). Social capital in the area of relationships is accentuated more in the statements of teachers working in primary school in grades 1–3 (M = 16.14; SD = 14.57) than those working in secondary school and those working at different educational stages simultaneously (Table 4).

Statistically significant differences were also found in the magnitudes of social capital resources in the relationship domain between secondary school teachers and those working in primary school in grades 4–8 (W = 5.16729; *p* < 0.01), indicating that statements from the latter were more concerned with this aspect of social capital (M = 13.86; SD = 12.27) (Table 4).

There are also statistically significant differences in the amount of social capital resources in the area of involvement between pre-school teachers and those working in primary school in grades 1–3 (W = −4.1627, *p* < 0.05) and grades 4–6 (W = −5.0737, *p* < 0.01) as well as those working in secondary school (W = −5.0313; *p* < 0.01). Social capital in the area of engagement is more evident in the statements of pre-school teachers (M = 4.346; SD = 6.989) than those working in grades 1–3 (M = 2.832; SD = 6.154), grades 4–8 (M = 2.947; SD = 6.251), and those working in secondary school (M = 2.962; SD = 6.947) (Table 4).

There are statistically significant differences in the use of words expressing positive affect between teachers working in a secondary school and teachers working at more than one stage of education (W = 4.0985; *p* < 0.05). Words expressing positive affect were much less emphasized in the statements of female teachers working in secondary school (M = 2.424; SD = 6.131) than those working at more than one stage of education (M = 3.661; SD = 8.595) (Table 4).

## 4. Discussion

In discussing the results of this study, we first comment on the resulting picture of social capital as perceived by teachers (descriptive aim of the study) and then examine the differences between teachers at different levels of education in terms of statements concerning social capital and emotional tone (explanatory aim of the study).

Answering a question about how teachers described their positive work experiences in terms of different aspects of school social capital and the emotional tone, we have noted the following characteristics of the respondents’ statements about positive remote learning experiences. The relational resources and fulfilling commitments were most frequently mentioned among the sub-dimensions of social capital. In their statements, teachers referred more frequently to the social capital resources, in terms of relationships and fulfilment of obligations, than to trust and commitment. Therefore, it can be assumed that teachers put particular emphasis on those types of resources, and these are probably the source of strength used to work in new conditions. According to other research conducted in Poland, the most important challenges in education during the COVID-19 pandemic were changing the way of working and maintaining relations with students [41]. While the quality of the relationship may have deteriorated during the pandemic [42], Polish students assessed the relationship with teachers as good and noticed a deterioration mainly in their relationships with classmates [43]. At the same time, studies in the pedagogical and psychological literature have found that relations are the condition for the success of distance work and education [35,41]. In the current study, social capital in terms of relationships is associated positively with positive emotions and negatively with negative emotions, making it possible to perceive relationships as the most critical resource of social capital in distance education.

The teachers’ statements were supposed to refer to the positive aspects of distance education. While the question was formulated this way, negative statements prevailed. This may suggest that the situation of people working in schools was difficult for them for various reasons. Similar results have also been found in other studies. Despite the positive aspects perceived by teachers in Poland, such as increasing their own professional competencies [23,44], convictions about the nuisance of this situation (and above all about its negative impact on students) prevailed [23,45].

When answering the research question about differences between teachers working at different levels of education in terms of referring to social capital when talking about positive experiences, the research yielded significant but small differences in terms of the generalized social capital index as well as its specific dimensions such as relationships and commitment. The results indicate differences in teachers’ experiences concerning the stage of education at which they work. Teachers in secondary school, and therefore those working with teenagers, in comparison to those working with younger children, emphasize less their social capital resources. Their statements about situations and events that they assess positively during distance learning also used fewer words expressing positive emotions. This could be because, as shown by previous results, social capital can be an important source of happiness [46], and if its resources were small, the challenges of the COVID-19 pandemic could be assessed more negatively.

Our results indicate that teachers working in secondary school in Poland put less emphasis on building social capital during distance education, especially in terms of relationships, than do teachers working with younger children. Meanwhile, fostering relationships should be a priority for teachers working with youth during a pandemic. There is some evidence that school social capital plays a role in building adolescent well-being [47] and that building school social capital is an essential aspect of school health [3]. As the available studies show, community bonds and good interpersonal relations are significant for people in adolescence [48]. For example, it has been found that during the pandemic, satisfying relationships with peers, their support, and having friends were factors that reduced the negative effects of the experiences of that time on the functioning of young people [49]. The adverse effects of isolation can be minimized by maintaining good interpersonal relationships, as maintaining these relationships (also in a media-mediated form) is a factor that protects well-being [50,51]. The research also shows that the reasons for reduced motivation and commitment to learning during distance education were an increased sense of loneliness, decreased sense of belonging, and unresolved conflicts with friends [30,49,52,53]. During the COVID-19 pandemic, the literature regarding teachers, for example, in the report “Thinking about Pedagogy in an Unfolding Pandemic”, has strongly emphasized the importance of primarily caring for students’ well-being [53].

In our research, pre-school education teachers are mainly characterized by the fact that in their statements, they emphasized social capital more in terms of commitment and relationships than did teachers working with older children and youth. This is a very notable result. The available research reports during the period of distance education clearly show that this kind of education is the most difficult at the lowest stages [43,44]. Perhaps this is mainly due to organizational and technical difficulties [27]. According to the available Polish research, teachers were not adapted to distance learning because this form of work in Poland had not been used (especially in primary school and pre-school) [23,44]. Distance education for pre-school children requires additional support (for example from their parents) to participate in online classes. For teachers working in kindergarten, perhaps their emphasis on active participation and the need to motivate both themselves and parents to engage in joint activities was necessary for work to be possible at all. This might be why their resources of commitment and relationships were higher than others.

## 5. Conclusions

When describing situations and events that they viewed positively in remote education during the pandemic, teachers mainly referred to social capital resources in terms of relationships and fulfilling obligations in their statements. Building on school social capital (especially in terms of relationships) may be more difficult for teachers working with older students. Therefore, these teachers may need additional support and training on the importance and ways of building such resources in working with adolescents. These resources should be strengthened, and a school culture based on social capital in the area of relationships should be built.

## Figures and Tables

**Table 1:** Categories of social capital and emotional tone of statements with sample words (indicators).

Category	Dictionaries (Examples of Words Included in the Given Category)
Social capital	
Relations	EN: children, parents, students, contact, relationship, relationships, talks, group, groups, contacts, meeting, in contact, together, mutually […]
Trust	EN: they miss, support, help, assistance, appreciation, they appreciated, they help […]
Commitment	cooperation, collaboration, involvement, participation, interest, activities, active, willingly, they perform, they take, they want, actions, they participate […]
Fulfilling obligations	work, task, lesson, tasks, classes, lessons, teaching, activities, learning, I assess, they teach, teaching […]
Emotional tone	
Positive	positively, better, good, nice, support, well, appreciation, better, more willingly, they enjoy, fun, it enjoys […]
Negative	difficulties, problem, problems, trouble, difficult, lacking, tough […]

Note: Dictionaries were prepared for the Polish language but in the table we present the English translation.

**Table 2:** Descriptive statistics (*n* = 1211).

	M	SD	Skewness	Kurtosis	Shapiro-Wilk W	Shapiro-Wilk P
Generalized social capital	27.72	17.39	0.7496	1.187	0.9574	<0.001
Relations	13.38	12.11	1.621	4.258	0.8665	<0.001
Trust	1.44	4.255	5.628	46.95	0.3816	<0.001
Commitment	3.523	7.11	4.351	34.57	0.5482	<0.001
Fulfilling obligations	9.372	9.422	1.183	1.974	0.8748	<0.001
Positive emotional tone	3.21	6.844	4.663	39.87	0.5223	<0.001
Negative emotional tone	4.298	16.89	4.942	24.19	0.268	<0.001

**Table 3:** Different educational stages compared according to the social capital and emotional tone of statements (Kruskal-Wallis test).

	χ^2^	Df	*p*	ε^2^
Generalized social capital	27.940	4	<0.001	0.021459
Relations	35.556	4	<0.001	0.027309
Trust	5.006	4	0.287	0.027309
Commitment	22.016	4	<0.001	0.016909
Fulfilling obligations	5.702	4	0.223	0.004379
Positive emotional tone	9.601	4	0.048	0.007374
Negative emotional tone	1.610	4	0.807	0.001237

**Table 4:** Social capital and emotional tone of statements at different educational stages.

	Stages of Education at Which Teachers Have Worked				
	Nursery school/pre-school	Primary education grades 1–3	Primary education grades 4–8	Secondary education	Teaching in more than one stage
	Mean (SD)	Mean (SD)	Mean (SD)	Mean (SD)	Mean (SD)
Generalized social capital	29.71 (17.94)	28.78 (16.74)	28.17 (17.13)	22.10 (15.12)	27.03 (17.99)
Relations	14.30 (11.33)	16.14 (14.57)	13.86 (12.27)	9.898 (10.11)	11.95 (12.5)
Trust	1.538 (4.836)	1.420 (3.696)	1.553 (3.921)	0.8386 (2.494)	1.56 (4.97)
Commitment	4.346 (6.989)	2.832 (6.154)	2.947 (6.251)	2.962 (6.947)	3.76 (8.991)
Fulfilling obligations	9.523 (9.311)	8.388 (9.091)	9.804 (9.475)	8.403 (9.113)	9.75 (9.968)
Positive emotional tone	3.144 (5.781)	3.561 (7.278)	3.268 (6.967)	2.424 (6.131)	3.66 (8.595)
Negative emotional tone	4.525 (17.82)	4.608 (18.21)	4.285 (15.89)	4.925 (17.66)	3.18 (15.17)

## Data Availability

The data presented in this study are available on request from the corresponding author (S.J.). The data are not publicly available due to their containing information that could compromise the anonymity of research participants.
